# Persian version of the International ICD-11 Prolonged Grief Disorder Scale (IPGDS): Validity and reliability

**DOI:** 10.1017/S1478951524001901

**Published:** 2025-01-30

**Authors:** Shahab Yousefi, Fatemeh Abdoli, Ashouri Ahmad, Aliyaki Hajar

**Affiliations:** 1 Department of Clinical Psychology, School of Behavioral Sciences and Mental Health (Tehran Institute of Psychiatry), Iran University of Medical Sciences, Tehran, Iran; 2 Department of clinical psychology, University of Social Welfare and Rehabilitation Sciences, Tehran, Iran; 3 Department of clinical psychology, University of Social Welfare and Rehabilitation, Tehran, Iran

**Keywords:** Psychometric, IPGDS, bereavement, ICD-11, prolonged grief disorder

## Abstract

**Objectives:**

This study aimed to evaluate the psychometric properties of the Persian version of the International ICD-11 Prolonged Grief Disorder Scale (IPGDS).

**Methods:**

A total of 554 participants (18 years and older, 326 women) completed the Persian IPGDS along with other measures. Participants were recruited through convenience sampling. The study assessed confirmatory factor analysis (CFA), convergent validity, and reliability of the Persian IPGDS.

**Results:**

CFA supported a 4-dimensional model, indicating good structural validity of the Persian IPGDS. Convergent validity was established through correlations with measures of depression, anxiety, and PTSD.

**Significance of results:**

These findings suggest that the Persian IPGDS exhibits satisfactory psychometric properties, making it a valid tool for measuring Prolonged Grief Disorder (PGD) in Persian-speaking Iranian adults.

## Introduction

After losing a loved one, individuals at first encounter an intense grief response which is gradually relieved (Maciejewski et al. 2007) and do not reduce in intensity over time, manifesting in prolonged grief. According to the research, over time, these individuals are at greater risk for developing physical health problems such as cancer and heart disease (Lundorff et al.,^2017^; Ott 2003; Stroebe et al. 2008). Additionally, prolonged grief has been associated with an expanded chance of developing mental health conditions, including depression, anxiety, and posttraumatic stress disorder (Killikelly & Maercker, 2018; Shear et al. 2011). Accordingly, in 2018, following the proposal of the international workgroup (Maercker et al. 2013), the main symptoms of prolonged grief disorder (PGD) including preoccupation with the deceased, along with several signs such as emotional distress (anger, a feeling of guilt, and having disturbance in accepting the loss) and interference with daily activities, were introduced in the International Classification of Diseases-11 (ICD-11) for the first time (Killikelly et al. 2022).

It is worth noticing that the previously mentioned symptoms have to be endure for at least 6 months after the loss. As a disorder, nevertheless, prolonged grief has been initially examined in Europe and North America and the existent research basis for the validity and reliability of the diagnostic criteria and PGD prevalence have been developed by their researchers (Boelen et al. 2018; Maciejewski et al. 2016; Prigerson et al. 2009). Thus, the newest definition of PGD in ICD-11 is mainly based on the existing symptoms in the Western world; in addition, the diagnostic instructions are majorly appropriate for European, North American, and some Chinese cases (Killikelly et al. 2023).

Furthermore, it is vital to note that there is variability between diagnostic manuals regarding the duration of the grief experience needed before a diagnosis is considered. Some investigators argue that DSM diagnoses may be limited to the cultural context of American society (Aggarwal 2013; Littlewood 1996), and cultural contrasts within the involvement and expression of grief symptoms may not be fully captured in the current definition of ICD-11 (Killikelly et al. 2023; Stroebe & Schut, 1998). The grief prevalence, duration, intensity of response to the bereavement, and its consequences are different due to the existent differences in social norms and cultural expectations, and are directly affected by emotional attachment and cultural characteristics (Assare et al. 2014; First et al. 2011; Rubin et al. 2008). Owing to these individual and cultural differences, not only is it difficult to define the stages, duration, and complications of prolonged grief but also the prevalence and features of the mentioned disorder are not identical in various cultures and societies (Shear 2022). Consequently, to fill this cultural gap, DSM and ICD are searching for the cultural validity of each diagnosis (Killikelly and Maercker 2018) since there are several questions concerning how the symptoms of “normal grief” are distinctive from those of a “prolonged grief disorder” in different cultures around the globe (Killikelly and Maercker 2023).

In this study, therefore, the cultural considerations mean that the symptoms of PGD should be more acute, intense, serious, and longer than people’s cultural and religious context to diagnose this disorder (Killikelly and Maercker 2018). Furthermore, adopting a relativist perspective on disorder means the border between norm and abnormality is a social/cultural norm and the definition of disorder will be changed besed on specific cultural norms (Canino and Alegría 2008). Such a classification system can lead to the improvement of diagnosis and therapeutic communication as well as the enhancement of therapeutic results (Aggarwal 2013).

In ICD-11, it is crucial to consider the cultural characteristics for the tangible effect of social-cultural norms may not be always visible to therapists; hence, they should be ascertained before clinical diagnosis (Stelzer et al. 2020). The significance of understanding grief in cultural contexts lies in how these beliefs about grieving can influence key symptoms of grief-related disorders. Cultural norms shape people’s experiences of suffering, affecting the expression of behavioral problems, thoughts, and emotional distress (Kohrt et al. 2014). As an example, the Chinese bereaved parents suffering from prolonged grief exhibit severe physical symptoms (for example headache) and have functional disorders whereas the Swiss bereaved parents report more intense cognitive indications such as obsessive rumination (Xiu et al. 2016). Likewise, in a similar situation, the frequency of physical symptoms including neck and shoulder pain, headache, and digestive problems are increased in Japanese people (Shimizu et al. 2017). Moreover, 52 percent of the Cambodian refugees have reported on their dreams about the deceased which are accompanied by the increase of PGD symptoms (Hinton et al. 2013). Nonetheless, the larger part of the current criteria for PGD symptoms are emotional or cognitive; thus, they might be insufficient for understanding the experienced symptoms by Japanese, Chinese, Cambodian, or Iranian individuals (Killikelly et al. 2022).

Several research studies have indicated that an accurate diagnosis is crucial for providing effective and comprehensive treatment. (Yousefi et al. 2022), One key aspect of this is the availability of scales with strong psychometric properties. So the versatile International ICD-11 Prolonged Grief Disorder Scale (IPGDS) developed by C. Killikelly et al. (2020) made it possible. IPGDS is the first tool of prolonged grief based exclusively on the ICD-11 definition of PGD, additionally, it is the first measure to incorporate culturally relevant items of grief. IPGDS stands as a validated and reliable diagnostic instrument for identifying ICD-11 Prolonged Grief Disorder.

During the investigations carried out by the researchers, in 2 studies the psychometric properties of this scale were examined in the Iranian population. in 1 study its reliability was examined in a very small sample size (Neysi et al. 2023). In another study (Killikelly et al. 2023), researchers did not distinguish between Iranian and Turkish populations. Although there are similarities between the Iranian and Turkish populations, there are cultural and religious differences between these 2 nations. The most important difference between them is the Sunni religion in Turkey and the Shia religion in Iran, which has an impact on the beliefs related to grief. furthermore, the psychometric properties of this scale were not fully investigated.

Previous Persian studies have primarily utilized the translated PG-13-R (Ashouri et al. 2023), highlighting the need for formally validated ICD-11 PGD assessment tools in Iran. The objective of this study was to evaluate the psychometric characteristics of the Persian adaptation of the IPGDS among grieving Iranian adults, focusing on the factor structure, reliability, and other facets of validity.

## Method

This study was conducted in 2 distinct phases. The first phase involved the translation and adaptation of the IPGDS into Persian (as detailed in the “Translation and Adaptation Phase” section). The second phase focused on evaluating the Persian version of the IPGDS among bereaved participants.

### Translation and adaptation phase

Permission was obtained from Dr. Clare Killikelly prior to translating the scale into Persian. The IPGDS underwent translation into Persian in accordance with the guidelines for cross-cultural adaptation of self-report measures (Beaton et al. 2000). Two Persian bilingual individuals were initially engaged in the translation process. The first individual, a mental health professional well-versed in the subject matter, and the second individual, a professional translator lacking knowledge of the subject matter, independently translated the English text into Persian. Through a comparison of the 2 translations, an initial Persian version of the IPGDS was formulated.

Furthermore, 2 proficient translators, whose mother tongue was English but were also well-versed in IPGDS, translated the initial Persian version back into English. The research team, including 2 grief experts and the 4 translators, assessed both the original version and all translated versions. This collaborative effort resulted in the development of a draft version of the IPGDS in Persian.

To evaluate its clarity and readability, the initial edition of the IPGDS was given to 30 grieving adults. The outcomes of the initial implementation were examined by experts and translators, who made adjustments to the IPGDS items as needed. No modifications were proposed by the participants, enabling us to finalize the Persian version of the IPGDS.

### Participants and procedure

The study involved a total of 554 individuals. According to Kellar and Kelvin’s (2012) study sample guidelines, the minimum sample size required for conducting a factor analysis should be 5 to 10 times greater than the number of items in the instrument being used. The study recruited a total of 554 bereaved adults through convenience sampling to participate in the research. The first 277 participants were included in the EFA, while the remaining 277 participants were utilized for the confirmatory factor analysis (CFA) evaluation.

All individuals involved in the study were sourced via online recruitment methods such as social media platforms (WhatsApp, Instagram, Telegram, Facebook) and websites dedicated to online support groups for those who have experienced loss, as well as through advertisements on Google’s content network in Iran. After reviewing details about the research (such as its objectives and the confidentiality of participation) and providing informed consent, those who wished to take part were able to fill out various online questionnaires (including a sociodemographic information form, IPGDS, nine-item Patient Health Questionnaire (PHQ-9), Generalized Anxiety Disorder (GAD)-7, and PTSD Checklist for DSM-5 (PCL-5). The Persian version of the IPGDS, consisting of 33 items, was utilized. The completion of the entire process required approximately 21 minutes.

Throughout the collection of online data, various control measures were implemented. These measures included monitoring the duration it took for each participant to finish the questionnaire and checking if the questionnaire was submitted multiple times from the same computer. No factors were identified in the controls that could impact the data collection process’s reliability. Participants were provided with the researcher’s telephone number and email address for any issues encountered during the questionnaire completion. Data was gathered from October 20, 2022, to April 3, 2023.

The eligibility requirements consisted of 2 main inclusion criteria: individuals had to be above the age of 18 and have encountered the loss of a significant individual (such as a close family member, friend, or another person deemed important to the participant) within a timeframe ranging from 6 months to 10 years prior to the study. On the other hand, individuals who provided incomplete or inconsistent responses to all sections of the questionnaires were excluded from the study. It is important to note that no form of incentive was provided to encourage participation in the research measures.

### Sociodemographic information form

The questionnaire developed by the researchers contains details regarding the gender, age range, educational background, relationship to the deceased, reasons for the passing, and the duration since the bereavement of the participants.

### International ICD-11 Prolonged Grief Disorder Scale (IPGDS)

Prolonged grief symptoms were assessed using the newly developed IPGDS (Killikelly et al. 2020). Participants were asked to indicate the frequency of preoccupation, yearning, and emotional distress symptoms related to the loss of a loved one over the past month in the 13 items of the standard scale (IPGDS-13). This was done using a 5-point scale: 1 = almost never (less than once a month), 2 = rarely (monthly), 3 = sometimes (weekly), 4 = often (daily), and 5 = always (several times a day) (Killikelly and Maercker 2018). The cultural addendum (such as physical problems, impaired concentration, cry loudly, etc.) comprises an extra 20 elements derived from key informant interviews and has been verified in samples of German-speaking and Chinese individuals (Killikelly et al. 2020). The IPGDS was also shown to possess strong psychometric reliability and validity, with a high level of internal consistency (Cronbach’s α = .92), as well as strong convergent and criterion validity (refer to Killikelly et al. 2020).

### Patient Health Questionnaire-9

The PHQ-9, developed by Kroenke et al. in 2001, consists of 9 items and is specifically designed to identify major depressive disorder (MDD) in accordance with the criteria outlined in the 4th edition of the Diagnostic and Statistical Manual of Mental Disorders (DSM-IV) (American Psychiatric Association 1994). The internal consistency of the PHQ-9 was found to be excellent, as evidenced by a Cronbach’s α coefficient of .89 in the PHQ Primary Care Study conducted by Kroenke et al. (2001). Scores on the PHQ-9 are determined based on the frequency of specific feelings reported by the individual. Scoring involves assigning a value of 0 for responses indicating “not at all,” a value of 1 for responses indicating “several days,” a value of 2 for responses indicating “more than half the days,” and a value of 3 for responses indicating “nearly every day,” (Kroenke et al. 2001). Consequently, total scores on the PHQ-9 can range from 0 to 27, with higher scores indicative of more severe symptoms of MDD. Furthermore, the validity of the PHQ-9 has been established for use within the Iranian population, with a Cronbach’s α coefficient of .856 (Farrahi et al. 2021).

### The 7-item Generalized Anxiety Disorder scale (GAD-7)

The GAD-7 is a concise scale consisting of 7 items that individuals can administer to themselves. Its purpose is to identify the presence of Generalized Anxiety Disorder (GAD) and evaluate the severity of symptoms based on the criteria outlined in the DSM-IV (American Psychiatric Association 1994). This scale inquires about the frequency of anxiety symptoms experienced by respondents within the past 2 weeks. Each item is assigned a score on a 4-point Likert scale, ranging from 0 (not at all) to 3 (nearly every day), indicating the frequency of symptoms. The total score can range from 0 to 21, with higher scores indicating more severe symptoms of GAD. Based on the original validation studies, the total score can be interpreted as follows: no/minimal anxiety (0–4), mild anxiety (5–9), moderate anxiety (10–14), or severe anxiety (15–21). A cut-off score of 10 is suggested as a potential indication of GAD. In a study conducted with infertile individuals, the Persian version of GAD-7 demonstrated satisfactory psychometric properties (Omani-Samani et al. 2018). The study also found that the GAD-7 exhibited high internal consistency (α = .88).

### PTSD Checklist for DSM-5 (PCL-5)

The assessment of PTSD symptoms involved the utilization of the PCL-5. Individuals whose PCL-5 scores met the predetermined cutoff point underwent a clinical interview based on the DSM-5 criteria to establish a conclusive diagnosis (Blevins et al. 2015). Comprising of 20 items, the PCL-5 is a self-report measure where each item gauges the severity of a specific symptom on a 5-point Likert scale ranging from 0 (not at all) to 4 (extremely) over the previous month. This questionnaire is not limited to any particular event and can be employed in all types of disasters. Furthermore, it has demonstrated good validity and reliability in Iran (Sadeghi et al. 2016).

### Data analysis

Data were gathered through the utilization of Google Forms. The analysis of the data was conducted using SPSS software Version 26 and R Version 4.4.2. The reliability of IPGDS was assessed through Cronbach’s α and McDonald’s ω coefficients, mean inter-item correlations (MIIC), and test–retest reliability.

The test–retest reliability of the summed scores of the IPGDS items was assessed by analyzing data from 114 participants who took the IPGDS twice, with a 5-month gap. To evaluate the test–retest reliability of the IPGDS, we employed the intraclass correlation coefficient (ICC) using the 2-way mixed method. A Cronbach’s α and McDonald’s ω value exceeding .70 is considered acceptable (Raykov and Hancock 2005), while the optimal range for MIIC falls between .15 and .50.The MIIC is independent of the number of items, unlike Cronbach’s α, and therefore offers supplementary insights.

Prior to conducting the EFA, assess the normal distribution of the IPGDS data by examining skewness and kurtosis. We conducted 2 EFAs to investigate the underlying structure of the IPGDS. The first EFA included 33 items, excluding items 14 and 15, which pertain to cultural considerations and time since loss. We used maximum likelihood (ML) extraction with direct geomin rotation for this analysis. The second EFA focused on the 13 items specifically related to ICD-11 PGD symptoms. Additionally, CFA was employed to examine the factor structure of both the IPGDS and the ICD-11 PGD items separately.

To ensure the data followed a univariate normal distribution, we assessed the absolute kurtosis (<3) and skewness (<3) values. Hence, the CFA employed the ML estimator. The assessment of the results was conducted based on the chi-square value and other established model fit indices, namely RMSEA (root mean square error of approximation), CFI (comparative fit index), NFI (normed-fit index), and IFI (incremental fit index).

To determine a satisfactory model fit, the ratio of the chi-square value, which is influenced by the sample size, to the degrees of freedom (χ^2^/df) should fall within the range of 2–3. This range signifies a favorable model fit (Schermelleh-Engel et al. 2003). In addition, it is worth noting that fit indices such as CFI, NFI, and IFI can serve as indicators of acceptable fit, with values of .90 and above (Hair et al. 2010). On the other hand, values of .95 and above are considered to indicate a good fit (Hu and Bentler 1999). To establish the convergent validity of the IPGDS, including the ICD-11 PGD symptoms sum scores, person correlation tests were conducted with other measures of psychopathology, such as PHQ-9, GAD-7, and PCL-5 sum scores. Furthermore, known-groups validity t-tests and correlation analyses were performed to examine differences in severity levels of IPGDS and ICD-11 PGD, as well as background variables such as gender and educational level, and factors related to the deceased and the cause of loss.

## Results

### Characteristics of the sample

The sample consisted of 326 women (58.6%) and 228 men (41.4%). Table 1 provides a summary of the sociodemographic characteristics of the participants. The average IPGDS score for the participants was 91.43 (SD = 27.33), with a score range of 34–160. The mean item score was 2.76 (SD = .62). Analyzing the mean scores for each symptom item, it was observed that, on a group level, the participants scored higher on item 1 (longing/yearing) and the second highest on item 3 (sorrow). The lowest score was recorded for item 17 (the loss shattered my trust in life or faith in God), followed by item 16 (unhealthy behaviors).

**Table 1.** Sociodemographic and bereaved-related characteristics of the study sample

Variable		Frequency (%)
Gender	Women	326 (58.6)
	Men	228 (41.4)
Age of bereaved	18–30	61 (11)
	31–45	209 (38)
	46–60	223 (40)
	61–76	61 (11)
Level of education	Lower than university	304 (55)
	University	250 (45)
Cause of death	Natural cause (e.g., illness)	428 (77)
	Suicide	67 (12)
	Accident	48 (9)
	Homicide	11 (2)
Deceased relative is my …	Partner/spouse	107 (19)
	Child	121 (21)
	Parent	140 (27)
	Sibling	63 (11)
	Friends	35 (6)
	Extended family member	64 (12)
	Other	24 (4)
Time since loss	In years	5.17 (2.37)
IPGDS levels		91.43 (27.33)
ICD-11 PGD levels		39.38 (10.39)

### Factorial structure

Table 2 presents the data regarding the central tendency, kurtosis, skewness, and distributions of responses for each item of IPGDS. Subsequently, 2 EFAs were conducted. The first EFA comprised all 33 items, excluding items 14 and 15. The second EFA focused on the 13 items corresponding to the ICD-11 PGD criteria, with a sample size of 277. Before conducting the EFAs, the suitability of the data for factor analysis was assessed by calculating the Kaiser-Meyer-Olkin (KMO) measure of sampling adequacy and performing Bartlett’s test of sphericity. The KMO statistic, which ranges from 0 to 1, was used to determine if the data is suitable for factor analysis. Values greater than .6 are considered acceptable for factor analysis according to Kaiser (1974). Bartlett’s test of sphericity, on the other hand, determines if the correlation matrix significantly differs from an identity matrix. A significant result (*p* < .05) indicates that the data is appropriate for factor analysis. The KMO measure of sampling adequacy yielded estimates of .97 and .91 for the 2 combinations, respectively. Additionally, Bartlett’s tests of sphericity resulted in a significance level of *p* < .0001. These findings suggest that the correlations between variables were appropriate for conducting factor analysis.

**Table 2.** Item distribution of the IPGDS

statistics					Factor loadings in EFA after rotation					
					IPGDS				ICD-11 PGD	
Item	Mean	SD	Skewness	Kurtosis	Factor 1	Factor 2	Factor 3	Factor 4	Factor 1	Factor 2
1	4.11	0.95	0.76	−0.22		0.59				0.79
2	3.79	1.02	−0.47	0.53		0.70				0.78
3	3.99	0.99	0.54	−0.71		0.74				0.77
4	2.59	1.31	0.28	−1.02			0.65		0.47	
5	3.15	1.38	0.14	1.17			0.72		0.56	
6	2.49	1.34	0.37	−1.04	0.53					0.69
7	2.22	1.33	0.72	0.73−			0.68		0.45	
8	3.20	1.36	−0.19	−1.14		0.69			0.66	
9	2.89	1.24	0.35	−0.98	0.74				0.75	
10	2.81	1.32	0.54	1.14	0.71				0.77	
11	2.80	1.28	0.74−	−1.03	0.81				0.79	
12	2.78	1.21	0.12	−0.88	0.78				0.72	
13	2.56	1.31	0.31	−1.08				0.69	0.59	
14	2.74	1.29	0.17	−1.04	0.69					
15	2.24	1.16	0.70	−0.29		0.67				
16	1.75	1.12	1.47	1.27				0.67		
17	1.12	0.85	0.24	−1.37						
18	2.74	1.15	0.44	0.80				0.74		
19	2.81	1.34	0.98	−0.81		0.92				
20	2.40	1.27	0.50	0.85	0.86					
21	2.34	1.23	0.57	−0.64	0.79					
22	2.62	1.27	0.25	−0.97	0.83					
23	2.81	1.36	0.17	−1.12	0.79					
24	2.39	1.41	0.60	−0.93		0.82				
25	2.54	1.22	0.32	0.89	0.78					
26	2.05	1.16	0.89	0.11				0.80		
27	1.77	1.11	1.40	1.04		0.62				
28	2.96	1.40	−0.51	−1.25			0.81			
29	3.76	1.10	−0.54	−0.42		0.74				
30	3.38	1.44	−0.38	1.19			0.69			
31	2.89	1.25	0.58	−0.87		0.63				
32	2.81	1.18	0.18	−0.74		0.57				
33	2.33	1.26	0.57	−0.79			0.69			

In the initial EFA, 4 factors with eigenvalues exceeding 1.0 were identified. These 4 factors accounted for 60.45% of the total variance. Factor 1, named “adaptation difficulties,” consisted of 11 items and had an eigenvalue of 8.22, explaining 25.71% of the variance. Factor 2, identified as the “traumatic separation distress”, consisted of 11 items with an eigenvalue of 5.36, explaining 16.76% of the variance. Factor 3, known as the “emotional distress,” included 6 items with an eigenvalue of 3.10, explaining 9.70% of the variance. Factor 4, named the “functional impairment,” comprised 6 items with an eigenvalue of 2.65, explaining 8.28% of the variance. The 17th item did not meet the standard factor loading value of .3 in any of the factors, leading to its removal from the scale. In the second EFA, which incorporated ICD-11 PGD items, 2 factors were identified with eigenvalues exceeding 1.0. Factor 1, known as the “intense emotional pain,” consisted of 9 items with an eigenvalue of 4.13, explaining 31.82% of the variability. Factor 2, identified as the “separation distress” included 4 items with an eigenvalue of 2.96, explaining 22.79% of the variability.

### Structural validity

In order to evaluate the structural validity, a CFA was conducted to determine whether a single or multifactor structure explains IPGDS and the symptoms of ICD-11 PGD (*n* = 277). Apart from the 1-factor model, the 2-factor model and 4-factor model were also examined for IPGDS. Based on the results, the CFA findings revealed that the 4-factor model of IPGDS demonstrated a satisfactory fit to the data (See Table 3).The fit indices for the 1-factor model in ICD-11 PGD were deemed unsatisfactory, while the 2-factor model showed strong model fit (See Table 3). Consequently, we opted for the 2-factor model as the most suitable solution for ICD-11 PGD.

**Table 3.** Results of the confirmatory factor analysis

Models	χ^2^/df	RMSEA	CFI	NFI	IFI
IPGDS: 1-factor	4.89	0.09	0.82	0.80	0.82
IPGDS: 2-factor	3.77	0.08	0.83	0.81	0.83
IPGDS: 3-factor	3.64	0.07	0.85	0.83	0.85
IPGDS: 3-factor	2.78	0.05	0.84	0.91	0.93
PGD ICD-11 1-factor	4.72	0.05	0.83	0.81	0.83
PGD ICD-11 2-factor	1.97	0.05	0.96	0.94	0.96

RMSEA = root mean square error of approximation; CFI = comparative fit index; NFI = normed-fit index; IFI = incremental fit index.

### Internal consistency

The MIIC value was .54, ranging from .11 to .74, indicating a very good level of reliability. The highest MIIC value was observed between items 19 and 20 (0.74) (impaired concentration and feeling stuck in grief), followed by the second highest value between items 20 and 21 (inability to fall back into a rhythm) (0.70). On the other hand, the lowest MIIC value was found between items 4 and 18 (feel guilty and shattered trust in life or god) (0.11), with the second lowest value between items 6 (avoid reminders) and 18 (0.13). These findings demonstrate the excellent internal reliability of the IPGDS, as supported by the internal reliability estimates of McDonald’s ω and Cronbach’s α, which were both .96. Additionally, the internal reliability estimates of ICD-11 PGD were also good, as indicated in Table 4.

**Table 4.** Internal consistencies of IPGDS, ICD-11 PGD items and derived item combinations and correlations with the other indices of psychopathology

	Correlations			Internal reliabilities	
	PHQ − 9 scores	GAD − 7 scores	PCL − 5 scores	Cronbach’s ɑ	McDonald’s ω
Total IPGDS scores	0.57	0.53	0.66	0.96	0.96
IPGDS AD scores	0.53	0.49	0.52	0.92	0.92
IPGDS TSD scores	0.45	0.44	0.61	0.90	0.90
IPGDS ED scores	0.52	0.52	0.55	0.83	0.83
IPGDS FI scores	0.49	0.43	0.54	0.85	0.85
Total ICD − 11-PGD scores	0.47	0.46	0.60	0.88	0.88
ICD − 11-PGD IEP scores	0.43	0.41	0.57	0.86	0.86
ICD − 11-PGD SD scores	0.39	0.42	0.44	0.83	0.83

Abbreviations: IPGDS = International ICD-11 Prolonged Grief Disorder Scale; AD = adaptation difficulties; TSD = traumatic separation distress; ED = emotional distress; FI = functional impairment; IEP = intense emotional pain; SD = separation distress; PHQ-9 = nine-item Patient Health Questionnaire; GAD-7 = seven-item Generalized Anxiety Disorder Scale; PCL-5 = PTSD Checklist for DSM-5.

### Reliability

The IPGDS total score exhibited a favorable test–retest correlation (ICC = .863), while the ICD-11 PGD items also demonstrated good test–retest correlations (ICC = .832).

### Convergent validity

Specifically, the correlation between IPGDS and PCL-5, PHQ-9, and GAD-7 was determined to be .66, .57, and .53, respectively. These results provide evidence supporting the convergent validity of IPGDS. A comparable pattern of correlation coefficients was also observed for the combined scores of the 12 ICD-11 PGD items (See Table 4).

### Known-groups validity

The findings indicated that females, individuals with lower levels of education, and those who experienced the loss of a spouse or child (as opposed to another family member or close friend) due to suicide, accidents, or homicide (rather than natural causes) exhibited elevated total scores on the IPGDS and ICD-11 PGD measures (See Table 5).

**Table 5.** Sociodemographic and loss-related correlates of disturbed grief

	IPGDS	Test statistic	ICD-11 PGD	Test statistic
Gender	M(SD)	*t* = −6.621		*t* = −6.291
Men	76.22 (25.93)		33.89(10.24)	
Women	95.07 (26.42)		40.70(10.02)	
Education level,	M(SD)	*t* = −3.273		*t* = 3.532
Low	94.19 (26.69)		40.63(10.04)	
High	81.21 (26.46)		35.26(10.94)	
Relationship to the deceased	M(SD)	*t* = −6.741		*t* = −5.812
Other than spouse/ child	75.03 (23.92)		33.10(9.77)	
Spouse/ child	108.70 (24.98)		44.48(9.07)	
Cause of death	M(SD)	*t* = −3.633		*t* = −3.682
Natural	89.07 (27.03)		38.47(10.31)	
Unnatural	98.82 (27.06)		42.23(10.17)	

*Abbreviations*: IPGDS = International ICD-11 Prolonged Grief Disorder Scale; ICD-11 = 11th edition of the International Classification of Diseases; PGD = Prolonged Grief Disorder; ** *p* < .01.

## Discussion

The aim of this research was to assess the psychometric properties of the Persian edition of the IPGDS. The findings of this study indicate that the Persian version of the IPGDS is a dependable and valid measurement tool for assessing PGD in bereaved adults within Iranian culture.

The absence of feedback or suggested modifications from the bereaved participants in the initial evaluation of the IPGDS is noteworthy and may be attributed to the alignment of the Persian version of the IPGDS with their cultural and linguistic experiences. The clarity of the questions and attention to cultural nuances during the translation likely contributed to the acceptance of the instrument without the need for revisions. Furthermore, the structure and content of the IPGDS effectively captured grief-related experiences, resonating with the emotional and cognitive states of the participants. This demonstrates the validity and reliability of the instrument for assessing prolonged grief in Persian speakers. However, the lack of proposed changes does not imply universal applicability without adjustments. Future research should collect feedback from more diverse bereaved groups to further validate the IPGDS across various cultural and situational contexts.

The factor analysis results revealed that the data adhere to the 4-dimensional model, contradicting the findings of the Chinese and German versions of this scale, which suggest a 2-dimensional structure (Killikelly et al. 2020). In general, the results indicate that the 34-item Persian version of the IPGDS encompasses 4 factors. The first factor, labeled as “adaptation difficulties” primarily consists of items related to adaptation responses to loss and psychosocial functioning. The second factor, termed “traumatic separation distress” includes items reflecting the traumatic response to loss. The 3rd factor, referred to as “emotional distress” comprises items associated with the emotional consequences of loss. Finally, the 4th factor is labeled as “functional impairment.” The findings from factor analysis revealed that the ICD-11 PGD items follow a 2-dimensional model, in contrast to the Chinese and German versions of the scale, which suggest a unidimensional structure (Killikelly et al. 2020). The 2 factors identified in the ICD-11 PGD items are “intense emotional pain” and “separation distress.”

It is possible that cultural differences influenced the Persian translation of the IPGDS, especially in relation to item 17, which addressed “shattered trust in life or faith in God/a higher spiritual power.” The 17th item did not meet the standard factor loading value of .3 in any of the factors, leading to its removal from the scale. Moreover, due to Iran’s strong religious beliefs and the significant role of God in the mourning process, including this item might have been perceived as disrespectful toward these cultural and religious values. Therefore, it is important to consider the cultural context when interpreting the content of the scale. Further exploration through qualitative research may provide deeper insights into the cultural intricacies shaping the understanding and expression of grief among this population. Such investigations could illuminate the significance of certain items within the scale and enrich our comprehension of grief experiences across different cultural contexts.

Another purpose of this study was to examine differences between the Iranian and Turkish populations regarding grief psychology and related coping strategies and therapeutic interventions. As discussed in the introduction, we hypothesized that these differences would likely be influenced by the distinct religions, cultures, and customs of the 2 countries. The results showed that the Persian version of the IPGDS includes 4 factors, supporting this hypothesis.

The Persian version of the IPGDS demonstrated excellent internal consistency reliability with a Cronbach’s alpha of .96, consistent with findings in other countries (Killikelly et al. 2023, 2020). Despite the preference for Omega alpha total over Cronbach’s alpha due to its independence from sample size and number of items (Ashouri et al. 2023; Nasri et al. 2023; Revelle and Condon 2018), both measures yielded identical results in this study (0.96). The stability of scores over time was assessed using the ICC method. The test–retest correlation for the IPGDS total score was strong (ICC = .863, *p* < .01), as was the correlation for the ICD-11 PGD items (ICC = .832, *p* < .01). This study represents the first examination of the long-term test–retest reliability of the IPGDS, confirming the Persian version’s reliability.

Results indicated a positive correlation between the IPGDS and the PHQ-9, GAD-7, and PCL-5. The correlation between the IPGDS total score and symptoms of depression, anxiety, and PTSD aligns with previous research (Baş et al. 2022; Kokou-Kpolou et al. 2022; Lenferink et al. 2022). It was also found that prolonged grief shares similarities with depression but remains distinct from the disorder, consistent with prior studies (Spuij et al. 2012; Thimm et al. 2019; Yousefi et al. 2022). Boelen et al. (2010) explored symptoms of PGD, depression, and PTSD, concluding that PGD is a unique clinical entity (Boelen et al. 2010; Pohlkamp et al. 2018). Additionally, although anxiety is not a core symptom of PGD, the loss of a loved one can trigger anxiety, leading to prolonged grief, especially in cases involving significant challenges or hardships (Caycho-Rodríguez et al. 2021; Shear and Skritskaya 2012). While prolonged grief symptoms often coincide with depression and anxiety (Caycho-Rodríguez et al. 2021; Kokou-Kpolou et al. 2020), the correlations observed in this study suggest that these 3 symptom clusters represent distinct yet interconnected constructs (Boelen and van den Bout 2005; Dillen et al. 2009).

The findings from the known-group validity analysis revealed that individuals with lower levels of education, experiencing the loss of a child or spouse (compared to other types of relationships), being female, and dying in an unnatural manner (as opposed to a natural death) are associated with elevated levels of prolonged grief. These results align with previous studies and research that have identified the risk factors for PGD (Ashouri et al. 2023; Lenferink et al. 2022).

Certain limitations of this research necessitate acknowledgment. First limitation is a cross-sectional design versus longitudinal, particularly given grief can change over time. Another limitation, the sampling was carried out through a convenience method, thereby diminish the extent to which the findings can be generalized. Additionally, the study relied on self-report questionnaires and online platforms (WhatsApp, Instagram, Telegram, and Facebook), potentially introducing bias among participants. While self-report measures are valuable for assessing PGD, they have limitations. Response bias may occur due to social desirability or inaccurate recall. Subjective interpretation of experiences, influenced by mood and cultural norms, can also affect responses. Standardized measures may not capture the complexity of grief experiences, overlooking cultural nuances or unique aspects. While we acknowledge these limitations, future research should consider complementing self-report measures with other assessment methods for a more comprehensive understanding. First, the study solely relied on self-report scales to assess convergent validity, potentially leading to methodological bias. Self-report methods are subject to various biases such as social desirability bias, recall bias, and response bias, which can affect the accuracy and reliability of the data collected. It is crucial to acknowledge that the outcomes of this research may not be universally applicable to all grieving communities, especially those with distinct cultural beliefs and mourning practices. Further investigations are necessary to explore the cross-cultural reliability of the IPGDS and other tools for identifying PGD.

Nevertheless, this research marks the initial attempt to evaluate the psychometric properties of the IPGDS within the Iranian context. Noteworthy strengths encompass an almost equal distribution of genders among participants, a substantial community-based sample of individuals who have experienced loss, and a specific timeframe following the bereavement. Moreover, the sample includes individuals with varied profiles of loss, enhancing the potential generalizability of the findings to diverse grieving populations.

## Conclusion

The IPGDS, which is the first measure of prolonged grief based solely on the ICD-11 definition of PGD, also incorporates culturally relevant aspects of grief. The test–retest reliability of the IPGDS was found to be satisfactory, indicating its consistency over time in identifying bereaved individuals who may be at risk of developing PGD. The correlations between the total score of IPGDS and various combinations, as well as measures of psychopathology, provided supporting evidence for the instrument’s convergent validity. To summarize, the results of this study demonstrate that the Persian version of the IPGDS exhibits good reliability and validity.

## Data Availability

The datasets generated during and/or analyzed during the current study are available from the corresponding author on reasonable request.
